# Modeling hydrological processes and analyzing water balance utilizing remote sensing data and physical hydrological models in the Songnen Plain, China

**DOI:** 10.1371/journal.pone.0329816

**Published:** 2025-08-14

**Authors:** Zhong Lu, Jinliang Zhang, Chaoqun Li, Xi Wang, Guoping Lei, Kuo Li

**Affiliations:** 1 Yellow River Engineering Consulting Co., Ltd., Zhengzhou, Henan province, China; 2 Key Laboratory of Water Management and Water Security for Yellow river Basin of Ministry of Water resources, Zhengzhou, Henan province, China; 3 Henan Resources and Environment Survey Institute Co., Ltd., Zhengzhou, Henan province, China; 4 School of Humanities and Law, Northeastern University, Shenyang, Liaoning province, China; Sathyambama Institute of Science and Technology: Sathyabama Institute of Science and Technology (Deemed to be University), INDIA

## Abstract

Climate change and human activities have a substantial effect on the regional water cycle. An accurate simulation of the water cycle process in the Songnen Plain under the influence of climate change and human activities can aid in gaining a comprehensive understanding of the regional water cycle change pattern in a changing environment, which has significant scientific and practical value. Based on the MIKE SHE/MIKE 11 model, this study utilizes multi-source remote sensing data, measured hydrological data, and other basic data as data sources to simulate the water cycle process in the Songnen Plain over the past 40 years and analyze its pattern of change. The results show that groundwater level data derived from GRACE and GLDAS exhibits great accuracy in topographically varied regions and low accuracy in proximity to rivers. The estimated groundwater data and measured runoff data, integrated with the MIKE SHE/MIKE11 model, accurately replicate the changes in the water cycle within the Songnen Plain. Over the years, the Songnen Plain has experienced an average actual evapotranspiration of 421.61 mm, with an average rate of change of −0.36 mm/a. The average surface runoff has been 36.26 mm, with an average rate of change of −0.025 mm/a. The average groundwater level is 169.2 m, indicating a weak downward trend. The variations in the water balance of the Songnen Plain surplus and deficit throughout different time periods were 0.804 billion m³, 0.098 billion m³, −1.15 billion m³, and 0.645 billion m³, respectively. Regarding alterations in various land uses, water supply and demand are most pronounced in arid regions, where the water balance exhibits a trend of initial decline followed by an increase; paddy fields experienced a water deficit across different time periods, with the severity of the deficit intensifying; the water balance deficit of building land increases with economic growth; and the water balance of other land uses was contingent upon climatic conditions. This study provides novel ideas and methods for regional simulation of water cycle processes in regional water scarcity literature by innovatively integrating GRACE and GLDAS data with the MIKE SHE/MIKE 11 model.

## 1 Introduction

The terrestrial water cycle is an essential link between the Earth’s spheres and a significant source of freshwater resources, playing a crucial role in social stability and economic growth [1,2]. In recent years, as a result of human activities and global climate change, the terrestrial water cycle process has become progressively more complicated [3], with an increase in extreme climate events such as flooding, drought, and groundwater depletion [4]. A proper understanding of the water cycle process under the influence of climate change and human activities plays a crucial role in the conservation and rational use of water resources [5]. The “China Medium and Long-Term Science and Technology Development Plan (2006-2020)” also clearly indicates that the response of regional hydrological cycle processes to global climate change and human activities is the current research hotspot in the field of “global change and regional response” [6]. Therefore, accurate modeling and analysis of regional land surface hydrological processes are scientifically and practically significant.

Hydrological models are an indispensable tool for the study of the terrestrial water cycle’s mechanisms [7]. Through the simulation of a hydrological model, the process of the water cycle can be quantified at various stages to disclose the law of hydrological change [8], thereby laying the groundwork for rational use of water resources and further study [9]. For instance, hydrological models have been employed to precisely replicate the water cycle in basins including the western United States [10], Lake La Picassa in Argentina [11], the Seine Basin in France [12], the Nile Basin [13], and the Mekong Basin [14]. Additionally, hydrological models have been employed to investigate the effects of climate change and human activities on the water cycle process in the Heihe Basin [15], the Yellow River Basin [16], the Xiangjiang River Basin [17], and the Songnen Plain [18] in China. Previous studies have produced many linked surface-subsurface hydrological models that integrate unsaturated zone (UZ) and saturated zone (SZ) water flow to investigate the principles of the water cycle by conceptualizing surface and subsurface hydrological processes, respectively [19,20]. The models are categorized into two types based on coupling methods: explicitly coupled models (e.g., SWAT-MODFLOW [21], GSFLOW [22]) compute surface and subsurface modules sequentially [23], whereas implicitly coupled models (e.g., MIKE SHE [24], PIHM [25]) are resolved concurrently within a unified numerical framework [26]. Implicitly coupled models enhance physical consistency and accuracy in simulating surface-groundwater interaction processes by fully preserving the flow continuity equations and boundary conditions [27].

The MIKE SHE/MIKE 11 model, as a representative example of an implicitly coupled hydrological model based on physical processes, has been extensively utilized in the simulation of hydrological processes. In particular, MIKE SHE/MIKE 11 performs better for surface water, groundwater, and their interaction [28]. Nevertheless, sufficient hydrological measurements are required for the model’s data support. Without adequate hydrological data, model simulation results and the actual process are susceptible to the “distortion” phenomenon [29]. Due to advancements in remote sensing and computer technology, hydrological models are increasingly incorporating remote sensing products as new data sources [30]. For instance, the Gravity Recovery and Climate Experiment (GRACE) satellite and the Global Land Surface Data Assimilation System (GLDAS) allow direct observations of terrestrial water storage [31]. Numerous studies have evaluated the precision and applicability of GRACE and GLDAS data at various scales, demonstrating their utility in hydrological applications [32–36]. Less research, however, has been conducted on how to integrate the MIKE SHE/MIKE 11 hydrological model with GRACE and GLDAS data in order to simulate regional water cycle processes. Furthermore, in examining land water balance, earlier investigations have predominantly focused on water cycles like precipitation, evapotranspiration, and surface runoff, while groundwater components have received comparatively less attention. In summary, this study innovatively integrates the GRACE and GRACE data with the coupled surface-subsurface model (MIKE SHE/MIKE 11) to simulate the entire process of land circulation in the Songnen Plain. This approach encompasses the entire process of land circulation and conducts an innovative investigation of the water balance in the Songnen Plain. In comparison to analogous remote sensing and hydrological model integration techniques in prior research, its principal advantage resides in the profound coupling of the physical mechanism model with satellite observational data, facilitating seamless multi-scale integration from the regional scale (100–200 km resolution of GRACE/GLDAS) to the local hydrological process (meter resolution of MIKE SHE/MIKE 11), thereby markedly enhancing the physical consistency and local accuracy of groundwater dynamic simulations.

The Songnen Plain is a significant grain-producing region and one of the most water-stressed regions in China [37]. Utilization of water for agricultural irrigation, industrial, and domestic purposes has substantially depleted regional groundwater reserves over time [38]. Due to the annual expansion of the rice cultivation area and the corresponding increase in irrigation water demand, the supply-and-demand situation for water resources has worsened further [39]. Consequently, accurate modeling of variations in the water cycle of the Songnen Plain is essential for ensuring food security and stable social development. This study focuses on the Songnen Plain, a representative region, as the subject of investigation; utilizes GRACE and GLDAS data, along with measured groundwater levels and other foundational data, as sources; and employs the MIKE SHE/MIKE 11 model to simulate the comprehensive hydrological cycle of the Songnen Plain, while examining the evolution of the water balance based on the entire hydrological process within the watershed. The study employs a rigorous and unique technique, with its research process and findings offering references and recommendations for future inquiries.

The main objectives of this study can be categorized as follows: 1) validating the accuracy of simulated groundwater results based on GRACE and GLDAS data; 2) simulating regional hydrological processes for 1980–2020 using the MIKE SHE/MIKE 11 model based on GRACE and GLDAS data; 3) identifying the characteristics of changing surface-subsurface water trends at the grid scale of the Songnen Plain from 1980 to 2020 and analyzing the changing patterns of the hydrological cycle.

## 2 Materials and methods

### 2.1 Study area

**Fig 1:**
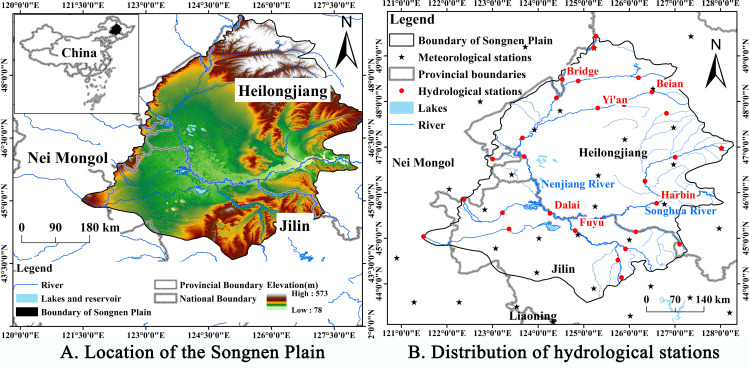
The topography and administrative division of the study area. (A) shows the specific distribution location of the Songnen Plain, located at the confluence of the Inner Mongolia Autonomous Region, Heilongjiang Province, and Jilin Province. (B) shows the distribution of groundwater monitoring stations in the Songnen Plain, which contains a total of 82 monitoring stations. Country and State basemaps source: National Earth System Science Data Center (https://www.geodata.cn/main/) [18,19], available under open license. Digital Elevation Model (DEM): Geospatial Data Cloud (https://www.gscloud.cn/search), freely available to use. Information on climatological and hydrological stations: Resource and Environmental Science Data Platform (https://www.resdc.cn/Default.aspx) [18], available under open license.

In this study, the boundary of the Songnen Plain was the China Geological Survey’s groundwater system boundary, which was drawn based on measured geological and hydrological conditions (Fig 1A). The Songnen Plain is one of the three major plains of northeast China, along with the Three Rivers Plain and the Liao River Plain, and is composed predominantly of the alluvial deposits of the Songhua and Nenjiang Rivers. The region is geographically situated between 121°38′-128°33′E and 42°49′-49°12′N, encompassing a total area of 18.28 × 10^4^ km^2^. The predominant climate type in the region is a temperate continental semi-humid and semi-arid monsoon climate. The region’s climate is distinct in every season due to the alternation of winter and summer monsoons. The average annual temperature tends to increase gradually from north to south, while annual precipitation ranges from 400 to 600 mm in the majority of regions and decreases gradually from east to west. As 60% to 70% of the region’s annual precipitation falls between June and August, it is susceptible to drought and inundation. The water system of the Songnen Plain reveals that the region is elevated on the periphery and low in the center. In the center of the Songnen Plain, the Nenjiang River, Songhua River, and their tributaries converge, forming a more concentrated river network. Moreover, 38 meteorological and 27 hydrological stations are located in the vicinity of the Songnen Plain (Fig 1B).

### 2.2 Groundwater storage change inversion model

#### 2.2.1 Inversion of groundwater storage changes.

To guarantee that the spatial resolution of the GRACE data is consistent with that of the GLDAS data, we have utilized the Raster package’s “aggregation” function [40]. The GRACE satellite data are expressed as changes in terrestrial water storage [34], whereas the GLDAS satellite data are expressed as the sum of canopy surface water changes, soil water content changes, and snow water equivalent changes [41]. From this, the following equation can be derived for the change in groundwater storage:


ΔGWS=ΔTWS−(ΔSM+ΔSWE+ΔCA) (1)

Where ⊿TWS represents the change in groundwater storage as determined by the GRACE and GLDAS data inversions, ⊿SM represents the change in canopy surface water, **⊿***SWE* represents the change in snow water equivalent, ⊿CA represents the change in soil water, and **⊿***GWS* represents the changes in groundwater reserves.

#### 2.2.2 Estimation of groundwater level changes.

This study employs the WTF method to estimate the change in groundwater level resulting from the inversion of groundwater storage variations. The fundamental principle of the WTF method employed here is that the groundwater storage anomaly is equal to the groundwater recharge over a lengthy period of time [34]. The specific formula is as follows:


$$\Delta h=\frac{\Delta GWS}{S_{y}}$$ (2)

Where ⊿**G*WS* represents the changes in groundwater reserves, *S*_*y*_ is the aquifer’s specified yield and ⊿*h* is the height of the groundwater change over a given interval of time.

#### 2.2.3 Preparation of model input data.

(1) Data from the GRACE satellite

The GRACE data selected for this paper is the GRACE Level3 RL06 product from the Jet Propulsion Laboratory (JPL) (obtained from https://podaac-tools.jpl.nasa.gov/drive/files/allData/tellus/L3/grace/land_mass/RL06). This product has a temporal resolution of months and a spatial grid size of 0.5° × 0.5°. Similar studies have demonstrated the usefulness of the data for groundwater storage inversion [42,43]. During the calculation process, the monthly data provided by JPL from January 2005 to December 2018 was selected, and the missing period was filled in by linear interpolation using the R function “approximate” [40]. All GRACE data were optimized to reduce leakage effects utilizing scale factors furnished by Mascon JPL.

(2) GLDAS data

The Global Land Data Assimilation System (GLDAS) is a data assimilation mission of the National Oceanic and Atmospheric Administration (NOAA) and the National Center for Environmental Prediction to simulate various hydrological and climatic variables, including four surface models: NOAH, CLM, MOSAIC, and VIC. In accordance with the recommendations of the Level 3 Data Product User Handbook [44], the GLDAS NOAH model with a 0.25° resolution was selected to estimate the total water content of the study area’s surface. The time periods for the employed data are identical to those of the GRACE data, and the necessary parameters for the simulated data are listed in Table 1.

**Table 1:** Details of features simulated by NOAH. Summarize information such as units for different parameters based on the NOAH model.

No.	Feature abbreviations	Name of the property	Units
1	canopint	total canopy water storage	kg/m^2^
2	qs	surface runoff	kg/m^2^/s
3	swe	Snow water equivalent	kg/m^2^
4	soilm1	0 ~ 10 cm average layer 1 soil moisture	kg/m^2^
5	soilm2	10 ~ 40 cm average layer 2 soil moisture	kg/m^2^
6	soilm3	40 ~ 100 cm average layer 3 soil moisture	kg/m^2^
7	soilm4	100 ~ 200 cm average layer 4 soil moisture	kg/m^2^

(3) Measured hydrologic data

**Fig 2:**
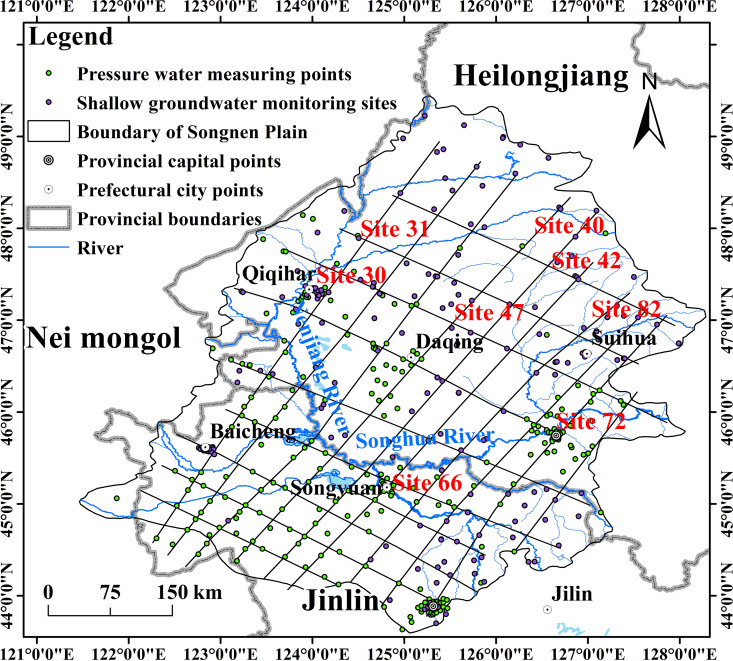
Distribution of hydrological and groundwater monitoring stations in the study area. Groundwater monitoring sites on the map include both bearing layer monitoring wells and potential layer monitoring wells. Information on hydrological stations: Resource and Environmental Science Data Platform (https://www.resdc.cn/Default.aspx), available under open license.

The measured groundwater data were provided by the People’s Republic of China in the “Yearbook of Groundwater Levels for Geological and Environmental Testing in China”, and the groundwater data between 2005 and 2018 were selected for this study. The total number of groundwater monitoring stations measured was 14,451, including 10,158 stations of the National Groundwater Monitoring Project (Ministry of Water Resources) and 4,293 stations of the National Groundwater Monitoring Project (Ministry of Natural Resources). There were a total of 433 groundwater monitoring stations in the survey area (Fig 2). Songliao Water Conservancy Network and Hydrological Observation Station collects information from 29 regional stations measuring surface runoff. This investigation utilizes effluent data from January 2005 to December 2018.

### 2.3 MIKE SHE/MIKE 11 model

#### 2.3.1 Model description.

**Fig 3:**
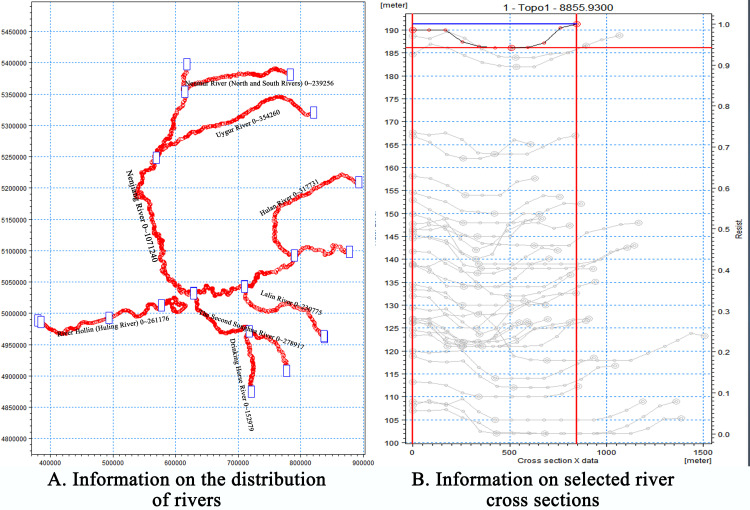
Hydrological information on the Songnen Plain. (A) presents the distribution characteristics of the water network in the Songnen Plain, where the blue boxes represent surface runoff monitoring stations. (B) presents the change characteristics of the Nenjiang River cross-section.

The MIKE SHE/MIKE 11 model has evolved from the SHE (Système Hydrologique Européen) model, which is a comprehensive distributed model based on a physical concept that allows simulation of the terrestrial phase of the hydrological cycle [45]. Evapotranspiration, surface flow, unsaturated zone, saturated zone, and channel flow are the model’s primary components. The dynamic coupling of MIKE SHE and MIKE 11 allows for the exchange of fluxes between the unsaturated zone, saturated zone, and surface flows. The evapotranspiration module of the model is computed using the Kristensen-Jensen method. The principal parameters required by this technique include crop coefficient (Kc), leaf area index (LAI), root depth (RD), and soil moisture content. Surface runoff is modeled using a two-dimensional finite-difference diffusion wave method. The entirely implicit finite difference decomposition of the one-dimensional Richard equation is used to represent the unsaturated zone, while the three-dimensional Darcy equation is used to simulate the flow in the saturation zone [46]. For the surface water flow and channel flow submodules, this study uses a simplified diffusive wave approximation of the Saint-Venant equation to define them, while for the saturated zone, the 3D-Boussinesq equation is used to simulate them. A simplified two-layer water balance method is employed for estimating the unsaturated zone, demonstrating effective performance in most instances [47]. The study region consists of the Songhua River and its network of tributaries (Fig 3A). Considering the size of the study area, DEM data extracted from ArcGIS were used to acquire river cross-section information (Fig 3B).

The integrated methodology of MIKE SHE with MIKE 11 employs three-dimensional variable saturated flow equations without explicitly differentiating the interaction between unsaturated and saturated zones. The dynamic coupling of MIKE SHE and MIKE 11 accounts for the interchange of water between the two models at every computational time step, with the majority of water exchange occurring in the river network line segment of MIKE 11. During the simulation, the river network traffic is transferred from MIKE 11 to MIKE SHE. MIKE SHE, in turn, estimates the slope flow and exchange volume between river aquifers in the network adjacent to each river link, and inputs the inflow or outflow of exchange volume in each time interval to the corresponding MIKE 11 river network. If the water level in the grid exceeds the topographical conditions, the area is deemed inundated. Once a square grid has been inundated, MIKE SHE calculates infiltration, seepage, slope flow, and evapotranspiration in the same manner as surface water accumulation [48].

#### 2.3.2 Model input data preparation and assumptions.

**Fig 4:**
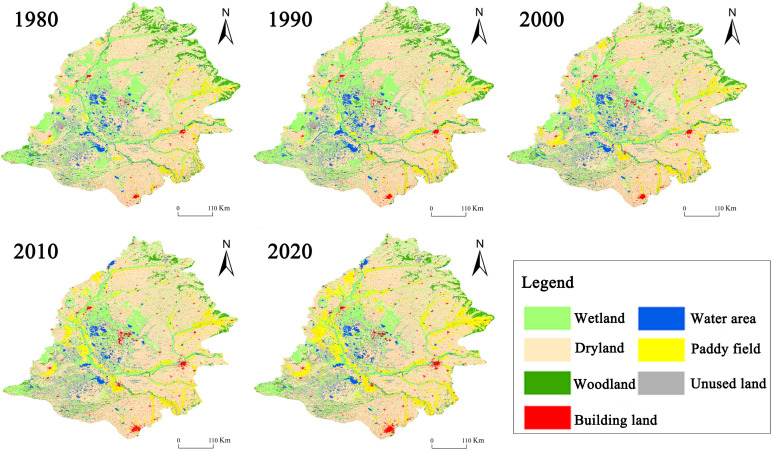
The spatial distribution pattern of land use/ coverage in Songnen Plain from 1980-2020. Based on Landsat satellite data, the land use types from 1980 to 2020 were interpreted to summarize the main land use types in the Songnen Plain. A detailed presentation of the regions with large land-use changes was also selected. Interpreted from landsat satellite data: USGS National Map Viewer (https://www.usgs.gov/tools/national-map-viewer), available under open license.

Fundamental data required for this simulation include soil attribute data, climate data, measured groundwater-runoff data, and river distribution data, etc., sourced primarily from the China Geological Survey, Songliao Water Conservancy Network, and China Science Data Center. From the USGS website’s Landsat data, 1980, 1990, 2000, 2010, and 2020 LUCC data were derived using a combination of ENVI supervised classification and ArcGIS visual interpretation (Fig 4).

**Fig 5:**
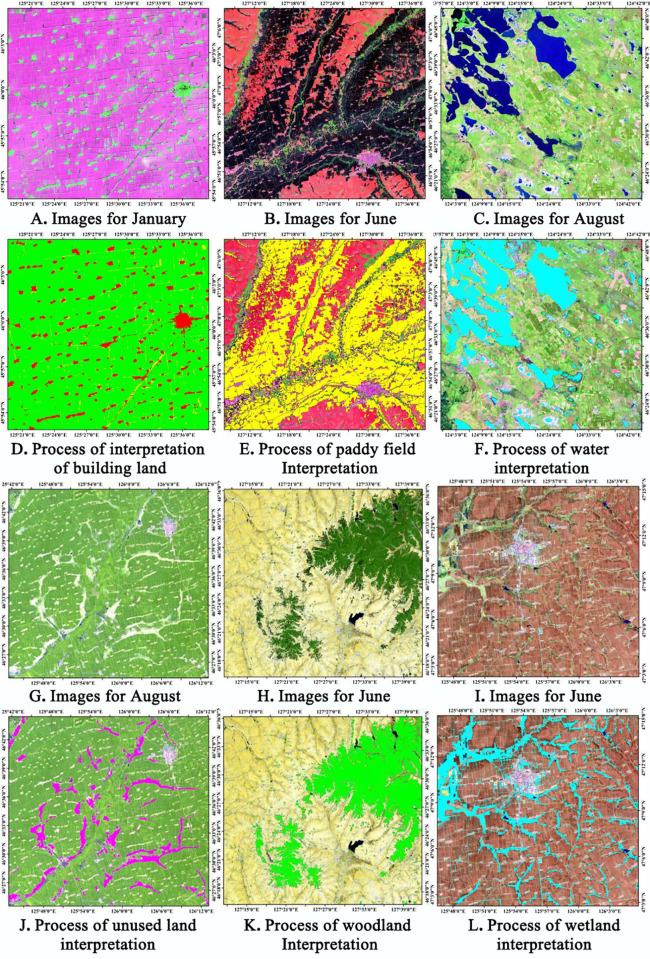
The remote sensing image features and interpretation results in different time periods. Figures A-C and G-I show Landsat image data, while Figures D-F and J-L show the distribution of interpreted areas. Landsat satellite data: USGS National Map Viewer (https://www.usgs.gov/tools/national-map-viewer), available under open license.

Using ArcGIS software, 500 sample sites in the Songnen Plain were selected at random in order to collect data regarding land use/land cover classification results in 1980, 1990, 2000, 2010, and 2020. The classification results were compared to the information provided by Google Earth using the Google Earth software. A confusion matrix was developed to evaluate the precision (Table 2). The results indicate that the method combining supervised classification and visual interpretation is appropriate for extracting land use/cover data. The average accuracy of the extracted data is 91.32 percent, and the aggregate kappa coefficient is 0.87. Based on the results of the above analyses, the land use data obtained from the analysis of this study demonstrated a high degree of accuracy, which is sufficient to meet the needs of subsequent studies. In order to improve the decoding accuracy, we extracted the data of different land use types based on the characteristics of remote sensing images in different months (Fig 5). Through the uncertainty analysis, it is found that there is a certain degree of uncertainty in the determination of the area of the watershed, which is mainly due to the fact that the watershed is significantly affected by precipitation, resulting in large fluctuations in its area. To integrate the land use data into the MIKE SHE/MIKE 11 model, this study employs Matlab software to convert the decoded raster data into DFS2 grid data that can be recognized by the MIKE SHE/MIKE 11 model. The file contains information on five phases of land use data. To accurately simulate the equilibrium between supply and demand in the Songnen Plain water cycle, the overestimation of water deficits in paddy fields was successfully mitigated by incorporating essential parameters such as conveyance efficiency (canal water use coefficient), field channel efficiency, and field application efficiency, grounded in the standardized treatment of water supply data for various land use types. The major channel has an efficiency of 94 percent, the secondary channel demonstrates an efficiency of 91.5 percent, and the field channel has an efficiency of 56 percent.

**Table 2:** Evaluation of LUCC classification accuracy in the study area. A confusion matrix was established to evaluate the decoded land use data, and the total accuracy and Kappa coefficient were counted.

Year	1980	1990	2000	2010	2020
Total accuracy (%)	93.71	90.26	91.82	89.43	93.16
Kappa coefficient	0.881	0.813	0.827	0.806	0.876

**Fig 6:**
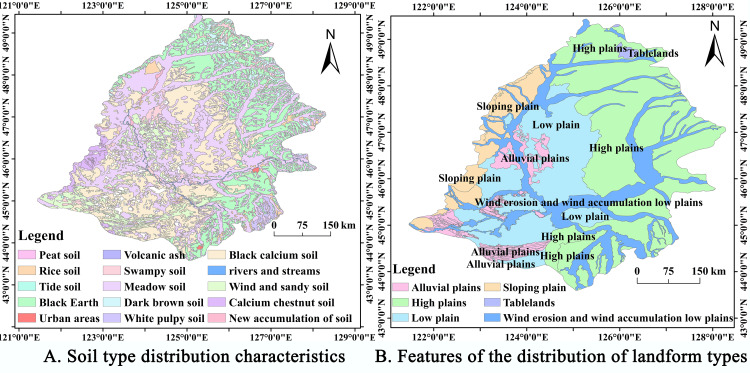
Map of the soils and landforms of the Songnen Plain. (A) represents the mapped soil property distribution characteristics. (B) represents mapped landform type data based on DEM data. Soil and terrain data: National Earth System Science Data Center (https://www.geodata.cn/main/), available under open license.

The Nanjing Institute of Soil Research, Chinese Academy of Sciences, provided soil data for the Songnen Plain from the China High Resolution National Soil Information Grid Basic Attribute Dataset (2010–2018) (Fig 6). NASA and METI provided DEM data for the Songnen Plain in the form of the ASTER Global Digital Elevation Model (ASTER GDEM). The horizontal accuracy of the DEM data is 1“(30m resolution, 95% confidence), and the resolution is 20m (95% confidence). Daily Values of Surface Climate Information in China (V3.0) is a precipitation dataset provided by the China Meteorological Network (http://data.cma.cn/). There are a total of 699 stations in the data, 38 of which are located within the scope of the study. Using the Tyson polygon method, we estimated regional precipitation [49]. Project information from the China Geological Survey included hydrogeological data (height of submerged groundwater floor, rock stratum distribution, rock properties, etc.). This study is constrained by a 0.3% spatial missing rate of soil and hydrogeological data, leading to elevation anomalies of ± 3.8 m in the digital elevation model, consistent with the maximum error threshold stipulated by national specifications. Additionally, the uneven spatial distribution of precipitation stations results in a relative error of 10% when applying Tyson’s polygonal interpolation method in sparse areas (with an absolute deviation of surface rainfall in a single month being <10 mm, adhering to the acceptable error threshold for hydrological forecasts). Nonetheless, the simulation results still fulfill the accuracy requirements of the study. The absolute deviation of one-month surface rainfall is less than 10 mm, satisfying the permissible error threshold for hydrological forecasting; nonetheless, the simulation result still fulfills the requisite research accuracy.

This study initially employed the WTF approach to get groundwater level data (TWSA) by integrating GRACE and GLDAS data for effective coupling with the MIKE SHE/MIKE 11 model. Subsequently, the 0.1° TWSA grid serves as the regional groundwater level constraint and is incorporated into the distributed hydrological model MIKE SHE/MIKE 11. The simulated groundwater level is aligned with satellite observations by modifying the soil hydraulic parameters and groundwater exchange coefficients. For the groundwater pumping module, the aquifer hydraulic conductivity and riverbed leakage coefficient are calibrated alongside the measured well bore water level data, and the effects of pumping activities on groundwater levels are quantified. The groundwater pumping module calibrates essential parameters, including aquifer hydraulic conductivity and riverbed leakage coefficient, using measured well bore water level data to assess the influence of pumping activities on groundwater levels. The final output of high-precision runoff and water level results is validated by Nash’s efficiency coefficient (greater than 0.8) and a relative error of less than 10%.

#### 2.3.3 Model sensitivity analysis.

This study employed the LH-OAT sensitivity analysis approach to choose sensitivity parameters, determining the model’s sensitivity by gradually altering one parameter while maintaining the others constant through numerous executions of the MIKE SHE model [46]. A sensitivity screening criterion of 0.05 was employed to ascertain the sensitivity parameters of the model, encompassing leaf area index, root depth, Manning’s roughness coefficient, wilting point water content, field water holding capacity, saturated water content, and saturated hydraulic conductivity. To guarantee the validity of the chosen parameter thresholds, this investigation was implemented in accordance with the parameter thresholds specified in the MIKE manual [50]. Parameter thresholds were established by referencing pertinent watershed studies in the region for parameters that are more pertinent to the study area [46] (Table 3).

**Table 3:** Ranking of parameters, model initialisation values and sensitivity of different components of the MIKE SHE/MIKE11 model. (1: most sensitive, NS: not sensitive).

Parameters	Range of values	Initial value	Sensitivity to water cycle impacts
**Slope flow parameter setting**			
Manning factor (M)	10-100	50	3
**Saturated zone**			
Horizontal hydraulic conductivity[m/s]	0.0012 - 1.8E-5	0.0001	1
Vertical hydraulic conductivity[m/s]	0.1*Kh		
Water production unit[-]	0.1 - 0.25	0.2	NS
Water storage unit[m^ − 1^]	1.00E-05	0.00001	7
**Unsaturated zone**			
Soil permeability[m/s]	1E-4 - 1E-7	1E-5	2
Field water holding capacity[-]	0.1 - 0.5	0.3	5
Saturated water content[-]	0.1 - 0.5	0.5	3
Water content at wilting[-]	0.01 - 0.2	0.1	4
Evaporation surface depth[m]	0.2-20	0.5	6

An analysis of model sensitivity reveals that the model is most sensitive to hydraulic conductivity, soil permeability, saturated water content, and Manning’s coefficient. Hydraulic conductivity controls the river’s baseflow and is a crucial subsurface factor affecting surface runoff. Permeability and water storage are the two most sensitive parameters for the unsaturated zone and water flow. These coefficients of scaling sensitivity are one magnitude greater than those of the remaining parameters. In terms of model parameter uncertainty, it is envisaged that hydraulic conductivity and recharge will have the greatest influence on the outcomes. The distribution of root depth is both a sensitive water table parameter and a land-use parameter. In such irrigated regions, root depth, which determines how much water is retained in the root zone, plays a crucial role in groundwater recharge. Additionally, ET depth and quiescent storage (a surface flow parameter that controls the quantity of surface water recharge) impact groundwater levels.

#### 2.3.4 Model calibration and validation.

This work utilizes observed runoff along with GRACE and GLDAS data to estimate groundwater level data for model calibration and validation. The primary principles for selecting estimated groundwater level data are that its change rule is consistent with the measured groundwater data, and the two exhibit a significant degree of similarity. The model is calibrated and verified by importing this data as groundwater site data. To calibrate the model, the Autocal tool of the MIKE SHE model was used. AutoCAL utilizes the Shuffle Complex Evolution (SCE) optimization algorithm, a global optimization algorithm that integrates a variety of search strategies, such as (i) competitive evolution, (ii) controlled random search, (iii) simplex morphology, and (iv) complex scrambling [37].

Utilizing the current hydrometeorological data and station information, the samples were allocated through the proportional distribution approach, assigning 70% of the most recent data to the training set and 30% to the test set. The entire monitoring sample size comprises 433, with 300 allocated to the calibration set for parameter optimization and 133 designated for the validation set to independently assess performance. The projected values from 1980 to 2004 and from 2019 to 2020. According to the data from 1980 to 2020, the extreme years for drought and flood identified by the percentile technique were 2003 (drought) and 2016 (flood), indicating that the validation period encompassed extreme events, and the routine validation inherently suggested exceptional conditions. Throughout the calibration procedure, the model was executed multiple times with varying parameter values until a suitable objective function was obtained. Several quantitative methods (objective functions) are used to evaluate model performance, including mean error, correlation coefficient, root mean square error, and Nash-Sutcliffe efficiency (NSE). The calibration targets used to compare observed and modeled data on a daily timescale were ME < 0.5m, r > 0.5, and RMSE < 1m for groundwater levels, and NSE > 0.75 and *F*_*Bal*_ for river discharge data, based on the scope and purpose of the model and previous regional studies with similar objectives [51].

### 2.4 Establishment of water equilibrium equations

The water equilibrium equation quantifies the extent to which the available water in a region satisfies its water demand, so efficiently assessing regional water balance. Based on relevant studies [52], this study takes into account the effective supply of water resources (including natural water supply, surface water recharge, and groundwater recharge) and the demand for water resources (domestic, industrial, agricultural, and ecological) and reflects the state of water balance in the Yellow River Basin in the form of a unit grid, which is calculated in the same way. The equation is as follows:


$$R_{i(t)}=S_{i(t)}-D_{i(t)}$$ (3)

Where: *R*_*i(t)*_ is the water balance index of land use type *i* in the equilibrium time period *t*; *S*_*i(t)*_ is the effective supply of water resources of this land use type in the equilibrium time period *t*, i.e., the water resources obtained by deducting surface runoff from rainfall (green water) and irrigation water (blue water) in the generalized water (m^3^); *D*_*i(t)*_ is the water resource demand for this land resource in this equilibrium time period *t*, i.e., the total water demand (m^3^).

## 3 Results

### 3.1 Comparison of inverse GWS and Songnen Plain well data from 2005–2018

**Fig 7:**
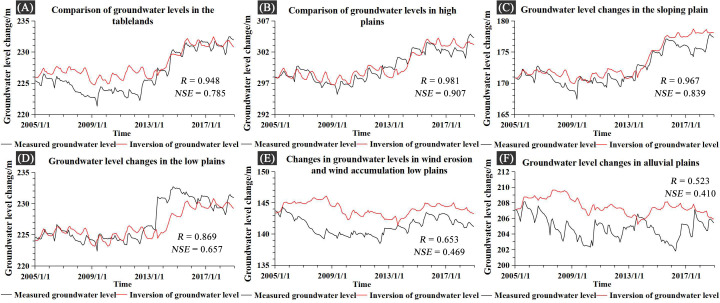
The comparison between the measured groundwater level data in different areas and the inversion groundwater level. (A-F) show a comparison between actual groundwater level data from monitoring stations in different regions and estimated groundwater level data. Trends in measured and estimated groundwater levels are summarized for different areas based on different geomorphological types. Measured groundwater level dataset: National Geological Library (https://www.ngac.cn/125cms/c/qggnew/index.htm), available under open license.

Groundwater levels estimated using GRACE and GLDAS data were calculated using the conversion relationship between changes in groundwater storage and groundwater levels. Mean changes in measured groundwater levels in areas with different geomorphologic types (Fig 6B) were counted against the corresponding mean changes in estimated groundwater levels. The results of comparing the differences between the two are shown below:

As depicted in Fig 7, the measured groundwater levels and remotely sensed inversions are in good agreement. For the high and sloping plains, the correlation between measured and remotely sensed inversion groundwater levels is very robust, with correlation coefficients above 0.95 for both. The gap between them is not evident. For the tablelands and low plains, there is a substantial correlation between measured and inverted groundwater levels, with average correlation coefficients at 0.948 and 0.869, respectively. However, there is a small discrepancy between the measured groundwater levels of the wells and the inversion-obtained groundwater levels. This is primarily due to the high spatial resolution of the inversion data, which represents the average groundwater level in a particular region and therefore introduces error. The correlation between actual and inverted groundwater levels is decreased for wind erosion, wind-deposited low plains, and alluvial plains, with average correlation coefficients of 0.653 and 0.523, respectively. This is primarily because this region is at a lower elevation. It is susceptible to lateral groundwater flow and fluvial influences, causing actual groundwater levels to be lower than inverted groundwater levels. The gap between the GRACE inversion and the actual groundwater measurements mostly results from the constraints of the temporal and spatial resolution of satellite data, as well as the delayed reaction of well water levels to aquifer recharge, resulting in systematic inaccuracies. In this study, in order to accurately validate and calibrate the MIKE SHE/MIKE11 model for the groundwater level data of remote sensing inversion, we primarily select those of high plains and sloping plains, followed by those of bit terraces and low plains, and we do not select groundwater level data from areas with wind erosion, wind-deposited low plains, and alluvial plains.

### 3.2 Model calibration and validation

#### 3.2.1 Calibration of model parameters.

**Fig 8:**
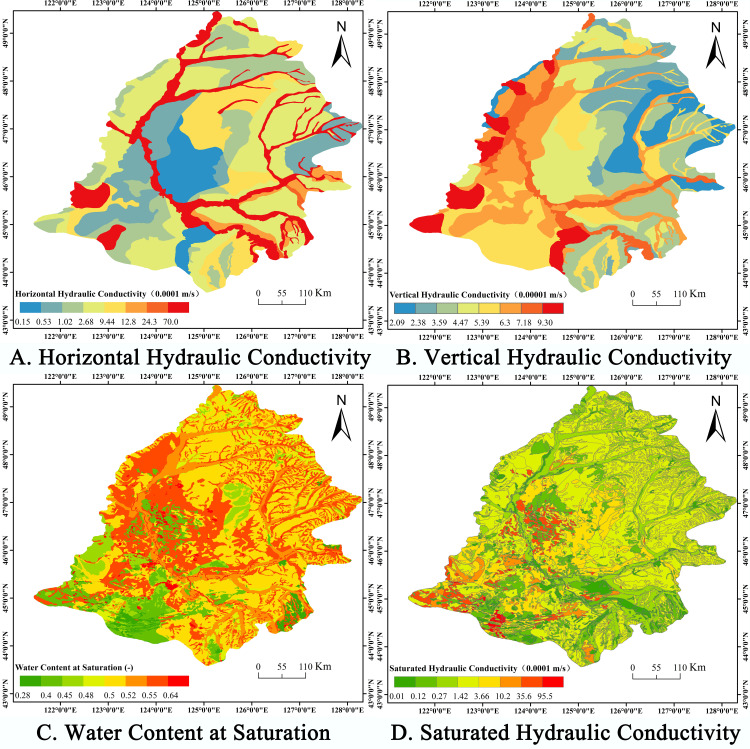
Results of parameter calibration on the Songnen Plain. (A-D) show a comparison between actual groundwater level data from monitoring stations in different regions and estimated groundwater level data. A combination of global search for excellence (SCE) and manual rate calibration was used to calibrate the parameter values for each of the four levels of groundwater calculation units.

Based on the varying sensitivities of the various parameters (Table 3), the Autocal automatic calibration procedure was used to calibrate parameters with high sensitivity and for which specific values could not be readily determined. To precisely calibrate the developed MIKE SHE/MIKE 11 model, the grid cells exhibiting correlation coefficients of 0.85 or higher between the groundwater level data derived from GRACE and GLDAS and the measured groundwater level data were chosen for calibration. In order to import the estimated groundwater levels from the GRACE and GLDAS data into the model, the centroid of each image in the estimated groundwater level raster data was assumed in this study to serve as the change in groundwater level in the area. Using Arcgis 10.2 software, the estimated groundwater level change raster data were converted to point data, and then the eligible point data were imported into the MIKE SHE/MIKE 11 model. In this way, the calibration and validation of the model were completed by importing the centroid of each eligible image and its groundwater level change characteristics into the model. For setting parameters that are less sensitive or for which specific parameter values are easily accessible, empirical or measured values were selected. In this investigation, the four groundwater calculation zones were used as the unit of calibration in order to more precisely calibrate the parameters. The parameter calibration results for each zone are presented below(Fig 8).

#### 3.2.2 Model calibration results.

**Fig 9:**
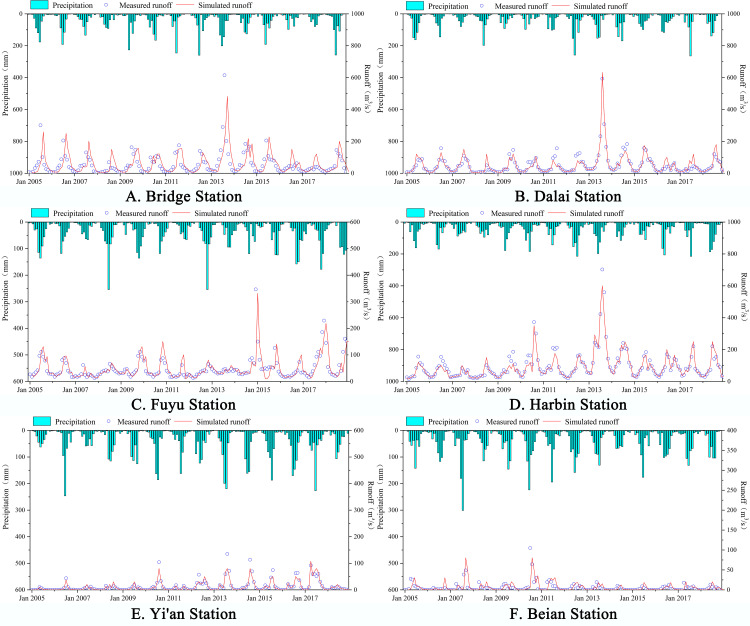
Comparison chart of actual runoff at each station and simulated value. (A-F) illustrate the comparison between the observed runoff at several hydrological stations and the simulated values. For the precise geographical positions of the various stations, please consult Fig 1B.

Monthly measurements of groundwater level, runoff, and soil moisture were used to calibrate the model using transient simulations. The mean periodic measurements were also used to perform spatial validation. Fig 9 and Table 4 depict the calibration results.

**Table 4:** MIKE SHE/MIKE 11 model calibration period and validation period model performance.

Hydrological stations	Calibration period (m^3^/s)				Validation period (m^3^/s)			
	Measured	Simulation	*NSE*	*F*_*Bal*_	Measured	Simulation	*NSE*	*F*_*Bal*_
Bridge Station	728.55	739.92	0.845	0.14	529.61	531.57	0.81	0.36
Dalai Station	655.90	651.64	0.836	0.2	488.46	479.50	0.82	0.46
Fuyu Station	636.78	644.77	0.812	0.15	531.47	538.76	0.81	0.36
Harbin Station	1222.32	1240.26	0.876	0.23	1048.57	1045.11	0.79	0.65
Yi’an Station	130.46	127.52	0.803	3.68	102.32	96.47	0.72	5.57
Beian Station	78.16	80.04	0.758	4.36	38.8	42.80	0.62	7.37
Average value	575.36	580.69	0.822	1.46	456.54	455.70	0.76	2.46

Fig 1B illustrates the precise locations of the runoff sites. The figure demonstrates that the MIKE SHE/MIKE 11 model provides an accurate simulation of the monthly runoff variability in the Songnen Plain, both for the rate period and the validation period. In comparison to the actual runoff, the simulated rise and fall processes correspond with the curves to a substantial extent. Nonetheless, the model tends to underestimate peak flood volumes (Fig 9). The simulation accuracy across various regions of the Songnen Plain (Fig 1) indicates that the middle and lower reaches of the Yellow River Basin exhibit superior accuracy compared to the upper reaches. This discrepancy is primarily attributable to the intricate topography and geomorphology of the upper reaches, coupled with the constraints of model parameterization accuracy.

According to Table 4, the Nash efficiency and water balance error coefficients for the calibration period are 0.822 and 1.46, while the coefficients for the validation period are 0.76 and 2.46. This suggests that the simulation results are superior, while the calibration period is marginally less effective than the validation period. On the basis of the F-test results, the model also passed the 0.01 significance test. Overall, the MIKE SHE/MIKE 11 model simulation accuracy is high.

**Fig 10:**
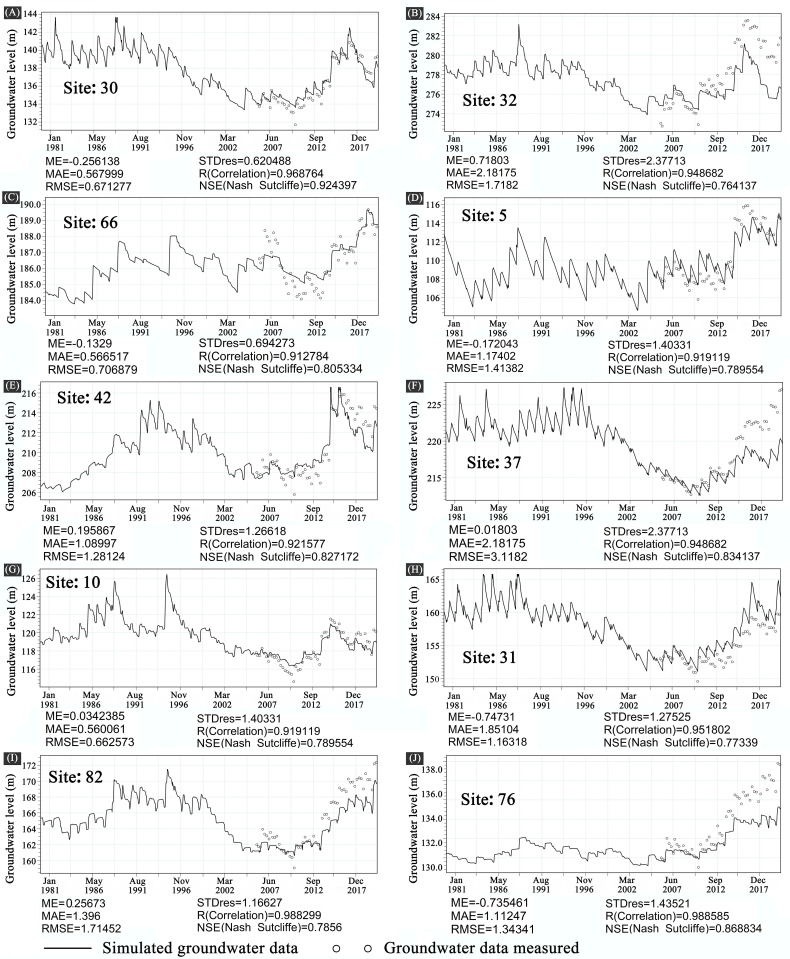
The comparison of actual groundwater level and simulated value at each site. (A-J) show a comparison between the measured groundwater level and the simulated groundwater level. Among the monitoring stations (Fig 2), 10 stations were randomly selected for calibration and validation of the presentation of results.

The MIKE SHE/MIKE 11 coupled model was calibrated and validated utilizing groundwater level data with high inversion accuracy. The calibration and verification outcomes of groundwater levels at select stations are illustrated in Fig 10. The precise positions of the randomly chosen sites are illustrated in Fig 2.

Comparison of the simulated groundwater levels with the inverse groundwater level time series reveals that a decent agreement can be attained for both the calibration and validation periods at various stations. The simulation accuracy of groundwater levels across various regions (Fig 2) indicates that the western segment of the Songnen Plain exhibits reduced accuracy. This is primarily attributable to its position at the lowest geomorphological elevation and the dense presence of wetlands, whose intricate hydrological interactions substantially heighten the uncertainty in groundwater dynamics simulation. Regarding the overall accuracy of the simulation, the correlation coefficients (R) and the Nash efficiency coefficients (NSE) are greater than 0.65 for the various sites. The Nash Efficiency Coefficient (NSE) is greater than 0.8 for the majority of the distinct sites (Fig 10). According to the hydrological model performance grading criteria proposed by Henriksen et al. (2003) [16], the goodness-of-fit of the model can be categorized as ‘excellent’ when the NSE value exceeds 0.75. The simulation results demonstrate that the MIKE SHE/MIKE 11 model accurately represents the groundwater flow mechanisms in the study area.

### 3.3 Analysis of water cycle processes

**Fig 11:**
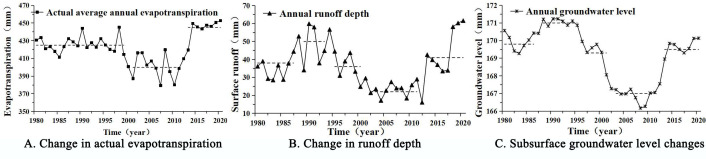
The interannual variation of runoff in Songnen Plain. Based on the simulated water cycle process, the change curves were plotted and clustered to analyze the change characteristics. (A) represents the actual evapotranspiration, (B) represents the surface runoff, and (C) represents the groundwater level. Information on climatological and hydrological stations: Resource and Environmental Science Data Platform (https://www.resdc.cn/Default.aspx), available under open license.

On the basis of the changes in the Songnen Plain’s water cycle between 1980 and 2020, as simulated by the MIKE SHE/MIKE 11 model, the mean values of the changes in the regional water cycle processes in various years were determined. The results are depicted in Fig 11.

According to the graph (Fig 11), the average annual actual evapotranspiration for the region is 421.61 mm. In 2020, the maximum annual actual evapotranspiration measured 452.83 mm. In 2007, the annual minimum actual evapotranspiration was 379.36 mm. During the study period, the actual evapotranspiration in the region decreased before increasing. Moreover, an analysis of the rate of increase in the region based on the Sen slope method yielded a rate of increase of 0.093 mm per year, indicating a weak upward trend in actual evapotranspiration over the past 40 years, which is primarily attributable to changes in regional temperature and precipitation. The region’s average annual runoff depth is 36.26 mm. In 2020, the greatest annual surface runoff is 61.57 mm. In 2012, the minimum annual surface runoff is 16.1 mm. Throughout the duration of the investigation, regional surface runoff exhibited a ‘increase - decrease - increase’ pattern, with surface runoff being primarily influenced by precipitation. The regional average groundwater level is 169.2 meters. 1999 marked the maximum regional groundwater level of 171.23 m. In 2009, the minimum groundwater level was 166.17 meters. The regional groundwater level exhibits a fluctuating pattern of “fall - rise - fall - rise” throughout the period, with a feeble downward trend.

**Fig 12:**
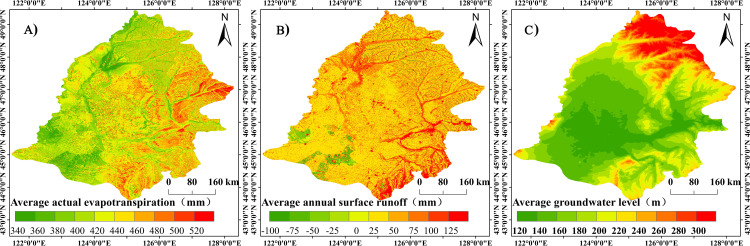
Characteristics of the distribution of different water cycle processes in the Songnen Plain. Characterization of the distribution of different water cycle processes using ArcgGIS. Where Fig 12A represents the actual evapotranspiration, Fig 12B represents the surface runoff, and Fig 12C represents the groundwater level. Country and State basemaps source: National Earth System Science Data Center (https://www.geodata.cn/main/), available under open license.

Fig 12 depicts the distribution characteristics of the various water cycle processes simulated by the MIKE SHE/MIKE 11 model. According to the graph, the average annual actual evapotranspiration of Songnen Plain ranges from 345.9 to 567 mm. The actual evapotranspiration increases gradually from the southwest to the northeast of the region in terms of spatial distribution characteristics. The majority of the regions with high actual evapotranspiration are located in Heihe City, the east-central region of Yichun City, and Harbin City’s central region. The majority of the regions with low actual evapotranspiration are to the west of Baicheng City, to the west of Qiqihar City, to the west of Daqing City, and to the north of Qiqihar City. The annual mean runoff ranges from −50.25 to 168.36 mm, and the spatial distribution pattern demonstrates a gradual decrease in potential evapotranspiration from the southwest to the northeast of the basin. The volume of runoff is particularly high on terraces, hills, and in the vicinity of highlands. The low values are primarily located near valleys and wetland areas, and runoff is also relatively low on the low plains. The average annual groundwater level varies from 98.12 to 365.68 mm. In terms of the distribution characteristics of groundwater levels, areas with high groundwater levels are primarily situated in regions with relatively elevated topography, whereas areas with low groundwater levels are primarily situated in the basin’s lower regions.

**Fig 13:**
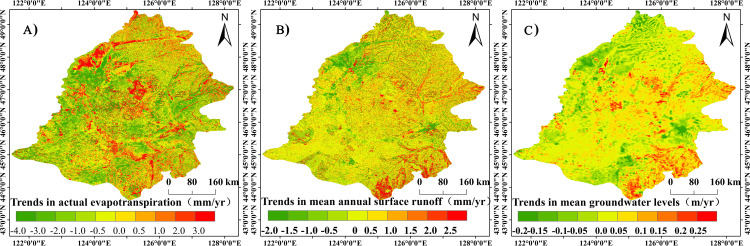
Spatial distribution characteristics of trends in various water cycle processes. Based on ArcGIS software, the evolutionary characteristics of different water cycle processes were mapped using image element trend analysis. Where Fig 13A represents the actual evapotranspiration, Fig 13B represents the surface runoff, and Fig 13C represents the groundwater level. Country and State basemaps source: National Earth System Science Data Center (https://www.geodata.cn/main/), available under open license.

Interannually, the trend in actual evapotranspiration on the Songnen Plain ranges from a decrease of −3.86 to an increase of 2.63 mm per year, with an average rate of change of 0.36 mm per year (Fig 13). In terms of the spatial distribution of the trend, actual evapotranspiration is relatively high in regions with abundant rainfall and relatively low in regions with arid land, unused land, and low rainfall. This is primarily because rice fields, wetlands, and water bodies are more abundant and susceptible to evaporation. The average annual rate of change for annual surface runoff is −0.025 mm per year, indicating a weakly declining trend. Surface runoff decreases in areas with more paddy fields, wetlands, and water facilities. The annual groundwater level exhibits a decreasing trend, with the majority of decreases occurring in higher terrain, near urban areas, and in regions with decreasing precipitation; the majority of increases occur in regions with increasing precipitation, expanding paddy fields, and water transfer from reservoirs.

### 3.4 Components of water cycle processes and their changes in characteristics

**Fig 14:**
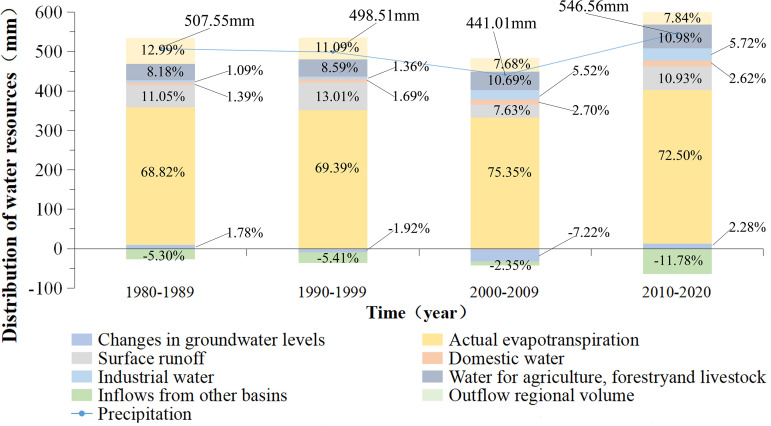
Characteristics of compositional shares of different water cycle processes in the Songnen Plain. The different water cycle processes have been simulated by the model of this study. Boundary data for the Songnen Plain: National Earth System Science Data Center (https://www.geodata.cn/main/), available under open license.

From 1980 to 2020, the water cycle process in the Songnen Plain was simulated using a calibrated MIKE SHE/MIKE 11 model. In various years, the characteristics of water cycle processes such as precipitation, actual evapotranspiration, runoff, and groundwater storage changes were measured. The results are displayed in the graph below (Fig 14).

As depicted in the graph (Fig 14), the long-term average precipitation in the Songnen Plain demonstrates a decreasing and then rising trend. Among the distribution characteristics of the surface water cycle process, actual evapotranspiration accounts for about 70% of the water cycle process in the region, followed by surface runoff accounting for about 10%, changes in groundwater storage accounting for about 5%, domestic water, industrial water, water for agriculture, forestry, livestock, and fisheries accounting for about 10%, and inflow/outflow of regional water accounting for 10%. Furthermore, the trends of changes in actual evapotranspiration and surface runoff are the same as those of changes in precipitation, both showing a decreasing trend before an increasing trend; the trends of changes in domestic water use, industrial water use, and water use for agriculture, forestry, and fisheries are increasing year by year; and the trends of changes in groundwater reserves are increasing. The quantity of water resources in the inflow region is gradually increasing, while the quantity of water resources in the discharge region is gradually decreasing.

### 3.5 Evaluation of water balance analysis results

**Fig 15:**
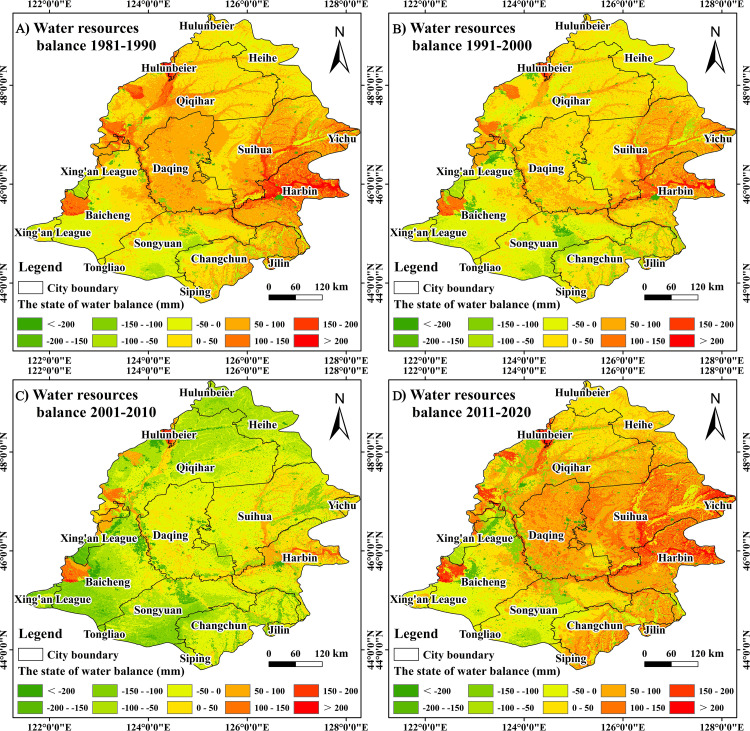
The spatial distribution of water balance in the Songnen Plain cell grid, 1980-2020. Based on the calibrated MIKE SHE/MIKE 11 model, the moisture equilibrium equation (Eq. 3) was used to find the distribution characteristics of the moisture balance in the Songnen Plain in different periods. Country and State basemaps source: National Earth System Science Data Center (https://www.geodata.cn/main/), available under open license.

Using the water balance equation, the water balance distribution characteristics were calculated for the entire area of the Songnen Plain (Fig 15), and the water balance status within different land use types was calculated (Table 5).

**Table 5:** The calculation of water balance in Songnen Plain, 1980-2020.

										(Unit: 10^8^m^3^)		
sub- basin	Water balance, 1981–1990			Water balance, 1991–2000			Water balance, 2001–2010			Water balance, 2011–2020		
	Su	De	Ba	Su	De	Ba	Su	De	Ba	Su	De	Ba
**Dryland**	28.4	24.1	**4.36**	27.3	25.1	**2.16**	20.8	25.1	**−4.30**	22.5	17.8	**4.72**
**Paddy field**	10.5	11.6	**−1.08**	11.8	14.3	**−2.5**	12.4	16.1	**−3.72**	16.7	19.2	**−2.5**
**Woodland**	8.4	8.08	**0.32**	7.3	7.17	**0.13**	5.2	5.5	**−0.30**	8.9	8.61	**0.29**
**Wetland**	6.1	2.75	**3.35**	5.3	4.01	**1.29**	4.8	5.56	**−0.76**	7.3	3.14	**4.16**
**Water area**	8.6	8.12	**0.48**	6.1	5.85	**0.25**	5	4.95	**0.05**	8.6	8.2	**0.4**
**Building land**	17.3	17.1	**0.25**	18.6	19.1	**−0.5**	19.5	21.6	**−2.13**	20.4	21.4	**−1.0**
**Unused land**	4.4	4.04	**0.36**	3.1	2.97	**0.13**	1.70	2.06	**−0.36**	2.5	2.09	**0.41**
**The total**	83.7	75.7	**8.04**	79.5	78.5	**0.98**	69.4	80.9	**−11.5**	86.9	80.5	**6.44**

Note: Su stands for the Water supply; De stands for the Water demand; Ba stands for the Water balance.

From 1980 to 2020, the water balance of the Songnen Plain exhibited a spatial trend of decreasing and then increasing, as illustrated in Fig 15. The number of cell grids in which the regional water volume decreased by more than 100 mm increased significantly when the regional groundwater storage variables from 1981 to 1990 were compared to those from 2011 to 2020. These grids were primarily located in the eastern part of Baicheng City, the northwestern part of Songyuan City, and the vicinity of Qiqihar City. It is evident that certain regions of the Songnen Plain are experiencing water consumption imbalance issues as a consequence of land use changes, even in the presence of abundant water. This necessitates the enhancement of water conservancy facilities, the optimization of the regional land use structure, and the mitigation of the trend of groundwater decline in the regional water resources imbalance zone. The decline in groundwater quantity is less apparent for positive values of regional water balance, suggesting that there is a surplus of water resources in these basins. This surplus can be further optimized to completely utilize the potential of water resources and enhance the efficiency of water use.

The water supply and demand for various land uses were aggregated to assess the water supply and demand balance features of several land uses(Table 5). Table 5 illustrates that the water balance in the Songnen Plain from 1981 to 2020 exhibited a pattern of decline followed by an increase, with changes quantified as 0.804 billion m³, 0.098 billion m³, −1.15 billion m³, and 0.645 billion m³, respectively. From various time periods, the dryland water balance status demonstrated a trend of diminishing and then increasing. The water balance was 0.436 billion m^3^, 0.216 billion m^3^, −0.43 billion m^3^, and 0.472 billion m^3^, respectively. The water balance of paddy fields during various time periods exhibited a deficit that intensified annually, with the water balance recorded at −0.108 billion m³, −0.246 billion m³, −0.372 billion m³, and −0.252 billion m³, respectively. The quantity of water balance in woodland land, wetland, and unused land exhibits a trend of increasing and then decreasing, primarily due to fluctuations in precipitation. In the context of construction land, economic development has led to a gradual deficit in the water balance of urban areas. However, the impact of human water transfer projects has mitigated this deficit. The water balance figures recorded were 0.025 billion m³, −0.052 billion m³, −0.213 billion m³, and −0.102 billion m³, respectively. Land use change, particularly the transformation of drylands into paddy fields, has led to a decline in the water balance (Ba) from a surplus of 8.04 during 1981–1990 to a deficit of −11.5 in 2001–2010, due to heightened water demand (De) and reduced infiltration. However, it partially rebounds to 6.44 in 2011–2020, indicating the beneficial impacts of irrigation optimization and ecological restoration.

## 4 Discussion

This study assesses the accuracy of changes in groundwater storage derived from the inversion by comparing changes in groundwater storage derived from GRACE and GLDAS data with measured groundwater data. Using precise regional groundwater inversion data and measured surface runoff data, the MIKE SHE/MIKE 11 model was used to simulate the water cycle in the Songnen Plain from 1980 to 2020. The results indicate that the results of the simulation are similar to the measured runoff and groundwater level data. For the model to simulate a lower runoff flood flow than the actual runoff, this is mainly because MIKE SHE/MIKE 11 does not take into account the effect of the accumulated temperature of the snow surface, and the amount of snowmelt is determined by the air temperature of the day, which makes the initial snowmelt fluctuate more with the air temperature rather than a smoother rising curve. In this study, the MIKE SHE/MIKE 11 model is utilized to achieve a coupled simulation of the surface-groundwater cycle by integrating GRACE-GLDAS multi-source satellite data with measured hydrological information, thereby enhancing the quantitative accuracy of runoff formation mechanisms and groundwater recharge pathways and offering a novel approach for investigating the water cycle process.

The GRACE satellite data has a geographical resolution of around 300–400 kilometers, constrained by the truncation of the spherical harmonic function order and orbital configuration, hence restricting their applicability to small and medium-sized areas [30]. To enhance resolution, researchers employ downscaling strategies, like random forest models or dynamical downscaling integrated with hydrological models; nevertheless, these methods may be influenced by data quality and regional peculiarities, resulting in additional mistakes. The uncertainties in GRACE data mostly encompass signal leakage, flaws in GIA models, and discrepancies among products from various organizations [31]. GLDAS data, however, encounters issues related to multi-source data fusion inaccuracies, structural model flaws, and temporal continuity challenges, resulting in subpar data quality, particularly in the arid regions of western China [34]. This study enhanced the accuracy of remote sensing satellite data by integrating GRACE and GLDAS data with the MIKE SHE/MIKE 11 hydrological model, achieving a precise simulation of the water cycle in the Songnen Plain. The model effectively captured groundwater flow dynamics in the arid western region (annual precipitation < 350 mm) due to significant evaporation and shallow groundwater interaction (Nash efficiency coefficient > 0.8). Conversely, in the eastern humid zone (annual precipitation > 600 mm), the model exhibited greater sensitivity to surface water (correlation coefficient R > 0.7) (Fig 10). This enables precise regulation of water resource management in the Songnen Plain, addresses groundwater overdraft, and advances the evolution of climate adaptation policies towards zonal planting, saline-alkali cycling, and coordinated interprovincial water resource scheduling.

Human activities and climate change are the main influences on the sustainable use of water resources. As a consequence of variations in precipitation, actual evapotranspiration, surface runoff, and groundwater levels fluctuate in response to climate change [53]. According to the findings of this study, the magnitude of the change in groundwater storage was greater than that of actual evapotranspiration and surface runoff, indicating that human activities are the primary cause of the decline in groundwater storage (Fig 14). Since the 1980s, groundwater extraction in the Songnen Plain has increased significantly, resulting in a downward trend in groundwater levels in the region [54]. The average groundwater level has decreased by 25 m in the regions of north-eastern Harbin, Suihua, Fuyu, and Qian’an, which are the principal areas of significant groundwater decline [55]. In addition, several significant overexploited areas, including Harbin, Daqing, Qiqihar, Suihua, Hailun, and Songyuan, exhibit varying degrees of groundwater depression cones [56,57]. All of these studies demonstrate that excessive groundwater extraction is the primary cause of the Songnen Plain’s persistent groundwater degradation. Compared with previous studies, the present study adopts a one-dimensional-two-dimensional coupled model (MIKE SHE/MIKE 11) and remote sensing data to accurately simulate the evolution characteristics of the groundwater level in the Songnen Plain, which is innovative to a certain extent. Moreover, the trend of groundwater level evolution is consistent with previous studies, which indicates the accuracy of the results of this study (Fig 13). This study confirms the model’s reliability and offers cross-methodological evidence for the conclusions regarding ongoing water table degradation, wetland reduction, and ecological risks stemming from groundwater over-exploitation, thereby augmenting the research findings value as scientific support for the formulation of water resource management strategies.

Several water conservation projects, including regional water transfer projects and the construction of water conservation dams, have been initiated in the Songnen Plain to mitigate the influence of human activities on groundwater reserves. The implementation of these initiatives has mitigated groundwater depletion in the region, with groundwater levels gradually recovering in Harbin, Changchun, and Daqing. In addition, the construction of water conservation structures has alleviated some of the agricultural water issues (Fig 13). However, the Songnen Plain’s current water resource management gives little attention to the connection between surface water and groundwater. The implementation of water resource initiatives, including dams, will alter the regional distribution of water resources. In the upstream areas, the construction of dams will increase subterranean groundwater levels, whereas in the downstream areas, underground groundwater levels are likely to decrease, which may further threaten the sustainable development of the regional ecosystem. For instance, the decline in groundwater levels in the downstream plains has resulted in a gradual increase in regional desertification [58], while the rise in groundwater levels in the upstream areas affected by the dam has resulted in an increase in regional soil salinization [59]. Compared with previous studies, the present study explores the evolution of the water balance in the Songnen Plain from the perspective of all elements of the water cycle. The main innovation is that the water cycle process takes into account the whole elements of surface water and groundwater, which leads to a higher accuracy of the estimated water balance state of the Songnen Plain. This study tackles the constraints of conventional approaches regarding temporal singularity and elemental fragmentation, markedly enhances the precision of water balance estimation, and offers a scientific foundation for the complementary utilization of water resources and ecological restoration. Land use change (LUCC) in the Songnen Plain substantially modifies the surface evapotranspiration framework (grassland to cropland to wetland/watershed) through a pattern characterized by ‘three increases and four decreases’ (increases in cropland, built-up land, and forestland, and decreases in grassland, unutilized land, wetland, and watershed). This transformation is primarily driven by the expansion of cropland, which increased by 10,490 km² from 1986 to 2020, alongside a reduction in wetlands, which decreased by 19,977 km², resulting in a notable alteration of the evapotranspiration structure (grassland to cropland increase of 46%). This markedly alters the dynamics of surface evapotranspiration (grassland to cultivated land elevates evapotranspiration by 46%), diminishes water conservation capacity, and interacts with climatic warming and aridity (precipitation rises by 10 mm per decade, and air temperature increases by +0.26°C per decade), resulting in a transition of the regional water balance from positive to negative, manifested as a reduction in surface runoff (forest to cultivated land raises runoff by 1–14.1%). Groundwater recharge diminishes (wetland degradation decreases infiltration rates by 30%), water deficit escalates during the growing season (evapotranspiration exceeds precipitation in 97% of the region, with an average deficit of 196 mm), ultimately causing a sustained decline in the water table (1–3 m) and an increase in salinization.

In addition, variations in regional actual evapotranspiration and surface runoff are sensitive to land use change, which has been shown to affect the substratum environment and, consequently, regional water cycle processes. This study found that the total proportion of actual evapotranspiration and surface runoff in the Songnen Plain increased from year to year as a consequence of land use change (Fig 14). This indicates that the utilization of water resources in the region is increasing year by year. This study innovatively integrates GRACE and GRACE data with a coupled surface-subsurface model (MIKE SHE/MIKE 11) to achieve the simulation of the whole process of land circulation in the Songnen Plain and deeply analyzes the state of the water balance evolution in the Songnen Plain, but there are still some uncertainties. The success of the simulation is ascribed to the high-precision filtering of GRACE data and the physical principles of the coupled model, whereas the failure or uncertainty primarily arises from parameter uncertainty caused by GRACE data noise, interpolation errors, and cognitive uncertainty due to the constraints of the MIKE SHE/MIKE 11 unidirectional coupling in low-lying areas and model simplification. In the future, uncertainty must be mitigated through dynamical downscaling, data assimilation, and structural optimization to enhance the adaptability of the coupled model in evolving contexts. How to increase the regional water use rate through regional land use planning and establish an agro-ecosystem that adapts to climate change is a hot topic and challenging area of research at the moment, and it will be the subject of future research in this study.

This study has three limitations: 1) Insufficient coverage of observation stations and missing historical data limit the completeness of verification for extreme hydrological events, potentially weakening the universality of extreme value inclusion; 2) The MIKE SHE/MIKE 11 model’s excessive simplification of hydrological processes reduces simulation accuracy for complex surface conditions with eastern-western heterogeneity; 3) Inherent noise in GRACE satellite data and coastal signal leakage effects reduce the reliability of quantitative water balance analysis, particularly when identifying subtle shifts between surplus and deficit states, which may introduce systematic errors. Future study will incorporate multi-source observational data assimilation and enhanced model coupling for optimization.

## 5 Conclusion

Based on GRACE and GLDAS data, this paper employs the distributed hydrological model MIKE SHE/MIKE 11 to simulate the regional water cycle process over the Songnen Plain. The main results are as follows:

The groundwater data derived from GRACE and GLDAS closely match the groundwater data that have been measured. Stronger correlations exist between measured groundwater levels and remotely sensed inversion groundwater levels in high and sloping plains. The relationship between terraces and low plains is strong. Wind erosion, wind-deposited low plains, and alluvial plains have a weaker correlation.

Based on the MIKE SHE/MIKE 11 model, the strongly correlated inverse groundwater data and actual measured runoff data were used to simulate the water cycle change process in the Songnen Plain, and the study demonstrated that the simulation accuracy was high.

The average annual actual evapotranspiration of the Songnen Plain is 421.61 mm, with an overall decreasing trend followed by an increasing trend; the high-value areas are primarily located in rice fields, wetlands, and regions with copious precipitation. The regional average multi-year surface runoff is 36.26 mm, with an increasing-decreasing-increasing trend; high-value areas are primarily located on unused land, in urban expansion areas, and in regions with increased precipitation. The average groundwater level in the region fluctuates between “decreasing-increasing-decreasing-increasing” with a weak decreasing trend throughout the period; the decreasing areas are primarily located in higher terrain, near metropolitan areas, and regions with declining precipitation.

The multi-year average precipitation in the Songnen Plain exhibits a decreasing trend preceding an increase. Based on the distribution characteristics of the surface water cycle process, actual regional evapotranspiration and surface runoff are the primary components of the water cycle process, and the trend of change is synchronous with precipitation. Changes in groundwater storage are influenced by precipitation and human activities, and the trend is an increase, followed by a decrease, and then an increase once more. The proportion of water used for human consumption, industry, agriculture, forestry, and fisheries accounts for approximately 10 percent of the water cycle and is increasing annually.

Between 1981 and 2020, the water balance of the Songnen Plain exhibited a pattern of decline followed by an increase, with variations in its water balance throughout distinct time intervals. The supply-demand balance of water resources in drylands was the most significant factor in the effects of various land use variations on the water balance of the Songnen Plain. The paddy fields exhibited water deficits at various intervals. The water balance of the construction land deteriorated progressively with economic development.
